# Assessing Post-traumatic Stress Disorder and Social Determinants Among COVID-19 Case-Contact Dyads in Household Settings

**DOI:** 10.7759/cureus.68425

**Published:** 2024-09-02

**Authors:** Aparna Ajay, Anas Tharakan, Aswathy Sreedevi, Lalithambika C. V.

**Affiliations:** 1 Community Medicine, Amrita Institute of Medical Sciences, Kochi, IND; 2 Physiology, Amrita Institute of Medical Sciences, Kochi, IND

**Keywords:** covid-19 survivors, covid-19 contacts, psychological impact, household contacts, ptsd

## Abstract

Background: Coronavirus disease 2019 (COVID-19) is a contagious disease caused by severe acute respiratory syndrome coronavirus 2 (SARS-CoV-2). The COVID-19 outbreak raised several public and mental health concerns including tremendous psychological distress.

Aim: To assess post-traumatic stress disorder (PTSD) among COVID-19-positive patients and household contacts and to determine the socio-demographic factors associated with PTSD in the study population.

Methods: A cross-sectional study was conducted among COVID-19-positive patients and their family members in Ernakulam district. A daily list of COVID-19-positive patients was obtained from the district officials. Confirmed cases and their close contacts in households were contacted over telephone and were interviewed after obtaining consent. Post-traumatic stress was assessed by the PTSD Symptom Scale - Interview Version (PSS-I-5) questionnaire.

Results: There were 279 study participants, of whom 93 were COVID-19 positive and the remaining 186 were their contacts. More than a third (34, 36.6%) of cases suffered from PTSD while about a fifth (40, 21.5%) of contacts suffered from PTSD. Among cases, persons belonging to the below poverty line (BPL) had 2.9l (1.19, 7.24) times higher risk compared to those above poverty line (APL). Women also had a 2.8 (1.14, 7.01) higher odds compared to males. Among contacts, graduates had a 9.54 (95% CI: 1.84, 49.36) increased odds whereas homemakers were found to be protected (0.195 (0.06, 0.66)) against PTSD compared to the employed group.

Conclusions: Psychological counselling and support are essential for addressing PTSD among women and those living with BPL, as these groups are disproportionately affected.

## Introduction

Coronavirus disease 2019 (COVID-19) is a contagious disease caused by severe acute respiratory syndrome coronavirus 2 (SARS-CoV-2). It has not only affected the physical health of humans [1] but has also caused psychological trauma among COVID-19 survivors and their household contacts [2]. The rapidly evolving pandemic has drastically altered people's lives and several aspects of the economy [3]. During the pandemic, patients with COVID-19 were more likely to experience stigmatization [4].

Exposure to infectious disease pandemics can cause traumatic experiences during treatment and confinement, as well as realistic or unrealistic fear of infection, social isolation, exclusion, and stigmatization [5]. A diagnosis of psychosis has been associated with viral exposure, treatments used to manage the infection, and psychosocial stress. Clinical management of these patients, where adherence to infection control procedures is paramount, is often challenging [6].

This, in turn, has had a significant impact on both personal well-being and societal health. To control the spread of COVID-19, most nations implemented measures such as social distancing and self-isolation. Even in areas without enforced restrictions, individuals remained anxious about contracting the virus [7]. While these measures were effective in controlling the disease [8], they also led to dramatic changes in daily life. Separation from loved ones particularly affected the mental well-being of COVID-19 patients, especially those who were severely ill, but conscious. The impact of these measures varied across pandemics and populations. For instance, Wang et al. [9] reported no immediate negative impact on University students. Additionally, individuals with social anxiety may have felt less anxious when staying at home, as this reduced the stress they typically experience from attending school or work [10]. Not only patients but also healthcare workers experienced mood and sleep disturbances during such outbreaks, underscoring the need to mitigate mental health risks [11]. It has been reported that merely experiencing or witnessing COVID-19-related suffering can lead to a high prevalence of post-traumatic stress disorder (PTSD) [12]. Therefore, it is not surprising that the relatives of COVID-19 patients experienced elevated stress levels.

PTSD is a common consequence of exposure to extremely stressful acute circumstances. According to the Diagnostic and Statistical Manual of Mental Disorders, Fifth Edition (DSM-5), the diagnostic criteria for PTSD include the following: exposure to actual or threatened death, serious injury, or sexual violence; the presence of intrusion symptoms related to the traumatic event; persistent avoidance of stimuli associated with the traumatic event; negative alterations in cognition and mood associated with the traumatic event(s), beginning or worsening after the event(s); marked alterations in arousal and reactivity associated with the traumatic event(s), beginning or worsening after the event(s); the duration of the disturbance; the disturbance causing clinically significant distress or impairment in social, occupational, or other important areas of functioning; and the disturbance not being attributable to the physiological effects of a substance (e.g. medication, alcohol) or another medical condition [13]. The risk of developing PTSD depends on an individual’s initial response to the traumatic event, the intensity of the memory, coping style, sense of safety, and social support [14].

Results from various studies across the world suggest that quarantine and isolation measures instituted to break the chain of transmission of COVID-19 were associated with an increased risk of post-traumatic stress [15]. Studies on young adults in the United States during the COVID-19 pandemic reported a PTSD prevalence of 31.8% [16]. A high proportion of individuals in the general population have also reported symptoms of anxiety (6.33% to 50.9%), depression (14.6% to 48.3%), PTSD (7% to 53.8%), psychological distress (34.43% to 38%), and stress (8.1% to 81.9%) during the COVID-19 pandemic in various countries such as China, Spain, Italy, Iran, the US, Turkey, Nepal, and Denmark [3]. The same systematic review identified risk factors associated with these distress measures, such as female gender, younger age (≤40 years), the presence of chronic or psychiatric illnesses, unemployment, student status, and frequent exposure to COVID-19-related social media and news [3].

The Indian government also implemented strict isolation measures and domestic quarantine policies, which helped reduce the spread of the disease, but these measures may have also resulted in adverse psychological effects. All forms of separation and isolation produce a certain amount of stress [17]. In Kerala, which mounted a strong response to COVID-19 through its robust health system, ward-level monitoring committees ensured that household contacts remained at home for 14 days and that index patients were isolated for 24 days or until their reverse transcription-polymerase chain reaction (RT-PCR) tests turned negative results [18]. These measures were in place during the period of this study and added to the stress associated with the disease. SARS-CoV-2-infected individuals, already burdened with psychological stress, experienced heightened stressors and struggled to cope adaptively with home isolation [19].

In a household context, social determinants, which are the proximal determinants, play a critical role. The presence of economic stability, employment, and social support has been found to reduce the risk of PTSD or symptoms of depression [20]. Social and economic circumstances significantly affect mental health [21].

There are no studies in India that have examined the impact of the COVID-19 pandemic on survivors and their contacts within a household setting. Exploring social determinants such as economic status, gender, and age is crucial for effective mitigation and future redressal. Flattening the caseload curve was essential for the population at large, but it came at a significant economic cost [22], affecting both individuals and families. The interaction between education, gender, and economic status is complex and should be examined within the context of COVID-19 transmission in households.

Therefore, this study was conducted to assess PTSD symptoms among COVID-positive patients and their household contacts, as well as the associated socio-demographic factors, using the PTSD Symptom Scale - Interview for DSM-5 (PSS-I-5) questionnaire.

## Materials and methods

This cross-sectional study was conducted among COVID-19-positive patients and their household contacts in Ernakulam district, Kerala. The study took place over a period of three months, from August to October 2021. Ethical clearance was obtained from the Institutional Ethics Committee of Amrita Institute of Medical Sciences.

COVID-19-positive patients from the designated hot spots in Ernakulam district were listed. The first two patients from the list were selected each day till 100 patients according to our inclusion and exclusion criteria were reached. Of the 100 patients who were approached, a few (seven) cited a lack of time and did not respond. The details of the study were explained to the participants and informed recorded consent was obtained. After obtaining consent, interviews were conducted over telephone between one to three months of COVID-19 diagnosis among the finalized consenting participants.

The sample size was calculated based on a study by Singh et al. [23], where the prevalence of PTSD was found to be 28.2%. Using the formula 4pq/d², with an absolute error of 10%, a sample size of 81 was obtained. For the case-contact dyad, cases were defined as RT-PCR or antigen-positive COVID-19 cases who provided consent or assent. Contacts were defined as all household members who had stayed in the same household for at least five days prior to testing positive. Exclusion criteria included cases where telephone communication was inappropriate due to dementia, learning disability, or other cognitive or communication impairments, as well as situations where there were no contacts for the positive patients or household members who were staying away from home during the incubation period of positive cases.

Information on sociodemographic variables was collected, and post-traumatic stress was assessed using the PSS-I-5 questionnaire. The PSS-I-5 is a 24-item semi-structured scale that assesses PTSD symptoms in the past month and makes a diagnostic determination based on DSM-5 criteria. Consistent with DSM-5, a PTSD diagnosis requires the presence of one intrusion symptom, one avoidance symptom, and two symptoms of clinically significant distress or interference, operationalized as a score of 2 or higher on the relevant items [24]. The scale in the local language, Malayalam, was provided by the PTSD National Center.

Participants' socioeconomic status was categorized as above the poverty line (APL) or below the poverty line (BPL) according to the color of their ration cards issued by the Government of Kerala. Those with white or blue ration cards were considered APL, while those with pink or yellow ration cards were classified as BPL. Educational status was divided into three categories: all with a high school education (at least eight years of schooling), higher secondary education (at least 12 years of schooling), and 15 or more years of education. Occupational status was categorized into employed, unemployed, and homemakers.

PTSD severity was considered the dependent variable. PTSD severity is determined by the sum of the 20 PSS-I-5 symptom ratings, with each question rated on a five-point scale: 0 for "Not at all," 1 for "Once per week or less/A little," 2 for "2 to 3 times per week/Somewhat," 3 for "4 to 5 times per week/A lot," and 4 for "6 or more times per week/Severe." Scores range from 0 to 80.

The clinical guidelines for PTSD symptom severity are as follows: 0-8 for minimal symptoms, 9-18 for mild, 19-30 for moderate, 31-45 for severe, and 46-80 for very severe. For the purposes of this study, participants with scores below 8 were considered not to have PTSD, while those with scores above 8 were classified as having PTSD, among both cases and their contacts [24].

Data were analyzed using the Statistical Package for the Social Sciences (IBM SPSS Statistics for Windows, IBM Corp., Version 20.0, Armonk, NY) [25]. Descriptive statistics were expressed as frequencies and proportions for categorical variables and as means and standard deviations for continuous variables. An univariate analysis was conducted to examine the association between PTSD in cases and contacts and selected variables. Multivariable logistic regression models were used to adjust for potential confounders. A p-value of < 0.05 was considered statistically significant.

## Results

We included 279 persons in the study of which 93 (33.3%) were COVID-19-positive patients and 186 (66.7%) were household contacts of these index (positive) patients. About 74 (39.8%) of the contacts later became positive during this study period. However, for the purposes of the study, we continued to analyze them as contacts. The mean age of participants was 36.4 ± 15.3 years. Among the cases, about half were 47 (50.5%) males (Table 1).

**Table 1:** Distribution of cases based on socio-demographic characteristics (N=93) BPL: below the poverty line; APL: above the poverty line

S. No.	Characteristic	Category	Frequency (N)	Percentage (%)
1	Age	≤36 years	54	58.1
		>36 years	39	41.9
2	Sex	Male	47	50.5
		Female	46	49.5
3	Residence	Rural	29	31.2
		Urban	64	68.8
4	Socioeconomic status	BPL	40	43
		APL	53	57
5	Education	High school	15	16.1
		Higher secondary	10	10.8
		Graduate	68	73.1
6	Occupation	Employed	81	87.1
		Homemaker	1	1.1
		Unemployed	11	11.8
7	Religion	Hindu	41	44.1
		Christian	34	36.6
		Muslim	18	19.4

Similarly, among the contacts, 101 (54.3%) were males (Table 2).

**Table 2:** Distribution of contacts based on socio-demographic characteristics (N=186) BPL: below the poverty line; APL: above the poverty line

S. No.	Characteristic	Category	Frequency (N)	Percentage (%)
1	Age	≤36 years	96	51.6
		>36 years	90	48.4
2	Sex	Male	101	54.3
		Female	85	45.7
3	Residence	Rural	58	31.2
		Urban	128	68.8
4	Socioeconomic status	BPL	80	43
		APL	106	57
5	Education	High school	27	14.5
		Higher secondary	61	32.8
		Graduate	98	52.7
6	Occupation	Employed	57	30.6
		Homemaker	100	53.8
		Unemployed	29	15.6
7	Religion	Hindu	82	44.1
		Christian	68	36.6
		Muslim	36	19.4

More than a third (34, 36.6%) of the index cases suffered from PTSD while only a fifth (40, 21.5%) of the contacts suffered from PTSD. Among the 279 cases and contacts, 74 (26.5%) suffered from PTSD.

Among the cases, about half 20 (50%) of those from the BPL category had a significantly higher proportion of PTSD compared to 14 (26.4%) of those from the APL group. This was reflected among the contacts also with 24 (30%) among the BPL category having PTSD compared to 16 (15.1%) among APL (Table 3).

**Table 3:** Univariate analysis to determine the association between various sociodemographic factors and PTSD in cases and contacts PTSD: post-traumatic-stress disorder; BPL: below the poverty line; APL: above the poverty line * A p-value < 0.05 is considered as statistically significant.

Variable	Cases		Odds Ratio	P-value*	Contacts		Odds Ratio	P-value*	
	No PTSD	PTSD			No PTSD	PTSD			
	(n=59)	(n=34)			(n=146)	(n=40)			
Age (in years)									
≤36 years	35 (64.8%)	19 (35.2%)	1.105	0.457	73 (76.0%)	23 (24%)	0.707	0.25	
>36 years	24 (61.5%)	15 (38.5%)			73 (81.1%)	17 (18.9%)			
Sex									
Male	35 (74.5%)	12 (25.5%)	4.982	0.022	79 (78.2%)	22 (21.8%)	0.01	0.53	
Female	24 (52.2%)	22 (47.8%)			67 (78.8%)	18 (21.2%)			
Residence									
Rural	19 (65.5%)	10 (34.5%)	0.78	0.484	53 (91.4%)	5 (8.6%)	8.28	0.002	
Urban	40 (62.5%)	24 (37.5%)			93 (72.7%)	35 (27.3%)			
Socioeconomic status									
BPL	20 (50.0%)	20 (50.0%)	5.467	0.017	56 (70.0%)	24 (30.0%)	6.001	0.012	
APL	39 (73.6%)	14 (26.4%)			90 (84.9%)	16 (15.1%)			
Education									
High school	50 (61.7%)	31 (38.3%)	1.088	0.581	25 (92.6%)	2 (7.4%)	7.02	0.03	
Higher secondary and above	9 (75%)	3 (25%)			121 (76.1%)	38 (23.89%)			
Occupation									
Employed	50 (61.7%)	31 (38.3%)	1.088	0.581	41 (71.9%)	16 (28.1%)	31.7	0	
others	9 (75%)	3 (25%)			105 (81.3%)	24 (18.6%)			
Religion									
Hindu	24 (58.5%)	17 (41.5%)	1.497	0.592	58 (70.7%)	24 (29.3%)	5.28	0.07	
Christian	22 (64.7%)	12 (35.3%)			58 (85.3%)	10 (14.7%)			
Muslim	13 (72.2%)	5 (27.8%)			30 (83.3%)	6 (16.7%)			

Among the cases, a significantly higher proportion of women (22, 47.8%) suffered from PTSD compared to males (12, 25.5%) though this was not seen in contacts. Among contacts, unemployed persons had significantly greater PTSD and PTSD appeared to be increasing with higher levels of education. As far as contacts were concerned, PTSD seemed to be more of an urban phenomenon with 35 (27.3%) suffering from PTSD compared to five (8.6%) among rural (p<0.002) (Table 3).

Following univariate analysis variables with a p-value of less than 0.2 were taken for multivariable logistic regression, and backward elimination was used to identify the significant predictors among the index cases and contacts. Among cases socioeconomic status and gender were independent determinants. Persons belonging to BPL had 2.9 (95% confidence interval (CI): 1.84, 49.36) times more PTSD compared to people belonging to APL. Women had a 2.8 times (95% CI: 1.14, 7.01) higher odds of PTSD compared to males.

Among the contacts, graduates had 9.54 (95% CI: 1.84, 49.36) times more PTSD compared to high school education. Interestingly, though unemployed people were found to have 2.72 times more PTSD compared to the employed group, this was not significant. However, homemakers seemed to be protected against PTSD (adjusted odds ratio (aOR) 0.19, 95% CI: 0.06, 0.66) (Table 4).

**Table 4:** Multivariable logistic regression to determine the independent predictors of PTSD among cases and contacts OR: odds ratio; CI: confidence interval * A p-value < 0.05 is considered as statistically significant.

Variable		Adjusted OR (95% CI)	P-value*
Cases			
Sex	Male	1	
	Female	2.828 (1.140, 7.011)	0.025
Socioeconomic status	APL	1	
	BPL	2.941 (1.193, 7.248)	0.019
Contacts			
Socioeconomic status	APL	1	
	BPL	1.203 (0.414, 3.498)	0.735
Education	High school	1	
	Higher secondary	2.721 (0.488, 15.167)	0.254
	Graduate	9.546 (1.846, 49.363)	0.007
Occupation	Employed	1	
	Homemaker	0.195 (0.058, 0.658)	0.008
	Unemployed	2.721 (0.911, 8.132)	0.073

## Discussion

In our study, PTSD affected more than one-third (34 cases, 36.6%, 95% CI: 26.8, 46.3) of COVID-positive index cases and about one-fifth (40 contacts, 21.5%, 95% CI: 15.5, 27.4) of their household contacts. Female gender was an independent determinant of PTSD among cases, with females being three times more prone to PTSD, similar to COVID-19 cases from BPL families. Among household contacts, higher education was associated with an increased risk of PTSD, while homemakers appeared to be protected from this risk.

A cross-sectional study conducted in Manipur among COVID-19 survivors identified PTSD symptoms in 67.5% of the 228 participants screened, highlighting the widespread nature of this mental health issue in the pandemic context [26]. In a study conducted in Saudi Arabia among children with a mean age of 12.25±3.77 years, symptoms of no, minimal, mild, and potential PTSD were identified in 15.5%, 44.1%, 27.4%, and 13.0% of children/adolescents, respectively [27]. Children who experienced isolation or quarantine due to COVID-19 were more susceptible to developing PTSD [28]. The prevalence of PTSD among adolescents ranged from 13% to 35.4%, with a notable 16.9% reported in high school students in a Chinese community after the lifting of strict lockdown measures [29,30]. Our study uniquely focused on the case-contact dyad, revealing a higher prevalence of PTSD in cases (34, 36.6%) compared to contacts (40, 21.5%). This contrast suggests varying degrees of psychological impact within these groups, which can be attributed to contacts witnessing the disease course in cases, allowing them to take proactive measures and be more mentally prepared.

Regarding PTSD symptom severity, an online survey conducted in the United States showed that moderate to severe symptoms were highest among adolescents (55%) [31]. Increasing age, employment in the private sector, urban residence, and referral by a doctor for COVID-19 testing were also significantly associated with PTSD symptoms [26]. Among healthcare workers in China, the prevalence of anxiety, depression, insomnia, and overall psychological problems during the COVID-19 pandemic was 46.04%, 44.37%, 28.75%, and 56.59%, respectively [32]. In a Korean study among patients diagnosed with COVID-19, 13 (20.3%) patients had a Posttraumatic Stress Disorder Checklist for DSM-5 (PCL-5) score of ≥33 [33].

Social determinants such as age, gender, residential area, socioeconomic status, education, occupation, and religion were assessed. COVID-19 cases from BPL families and contacts with higher education faced a higher proportion of PTSD compared to those from APL families and with lower education. Specifically, being from BPL families and having higher education among contacts were associated with increased risks of PTSD. A study from China also showed that vulnerable groups in the quarantined population were less financially well-off [34].

Female gender was an independent determinant of PTSD among cases, with females being three times more prone to PTSD. Other studies have also reported a higher vulnerability among women [35] and a doubled lifetime prevalence of PTSD compared to men [36].

Higher education among contacts was linked to a greater risk of PTSD. This suggests that education may play a complex role in influencing mental health outcomes in the pandemic context. Unemployment among contacts was associated with a higher incidence of PTSD, potentially linked to job and financial insecurity. This aligns with findings from Manipur, where individuals employed in the private sector exhibited more PTSD symptoms than their unemployed counterparts [26].

In a meta-analysis and systematic review [37], it was found that the risk of PTSD is higher following severe COVID-19 infection. PTSD symptoms were found to be more prevalent among COVID-19 survivors, healthcare workers, people living in constant fear due to close contact with infected individuals, and the general population affected by restrictive measures. Our study also found that COVID-19-positive patients suffered from PTSD more than the contacts.

Limitations

The study did not explore social support and structures in detail. However, it can be inferred that individuals with fewer resources, such as those from BPL categories and women, experienced more PTSD. Those from rural areas presumably had better support networks, leading to less distress.

## Conclusions

This study highlights the profound psychological impact of the COVID-19 pandemic on both infected individuals and their household contacts, with a particular focus on PTSD. The data reveals that women and individuals from BPL households exhibit higher susceptibility to PTSD, emphasizing the need for targeted mental health interventions for these vulnerable groups. Additionally, the heightened risk of PTSD among household contacts with higher education levels points to a complex interplay between awareness and stress. These findings underscore the importance of social determinants in influencing mental health outcomes during pandemics.

In summary, this study provides critical insights into the mental health challenges that surfaced during the COVID-19 pandemic, underscoring the necessity for comprehensive mental health strategies. Although the measures to control the spread of COVID-19 were essential, they also inadvertently contributed to psychological distress, particularly among vulnerable populations. Consequently, the urgent implementation of Psychological First Aid (PFA) - an evidence-based approach aimed at alleviating immediate post-disaster distress - is recommended, with particular emphasis on women, and individuals from lower socioeconomic backgrounds.
